# Supplementary material for: Doxorubicin Changes the Spatial Organization of the Genome around Active Promoters
Source: Cells. 2023 Aug 4;12(15):2001. doi: 10.3390/cells12152001 (PMC10417312; doi:10.3390/cells12152001)
Supplement: Supplementary file 1 [file cells-12-02001-s001.zip › cells-2530190-supplementary.pdf]

Supplementary information

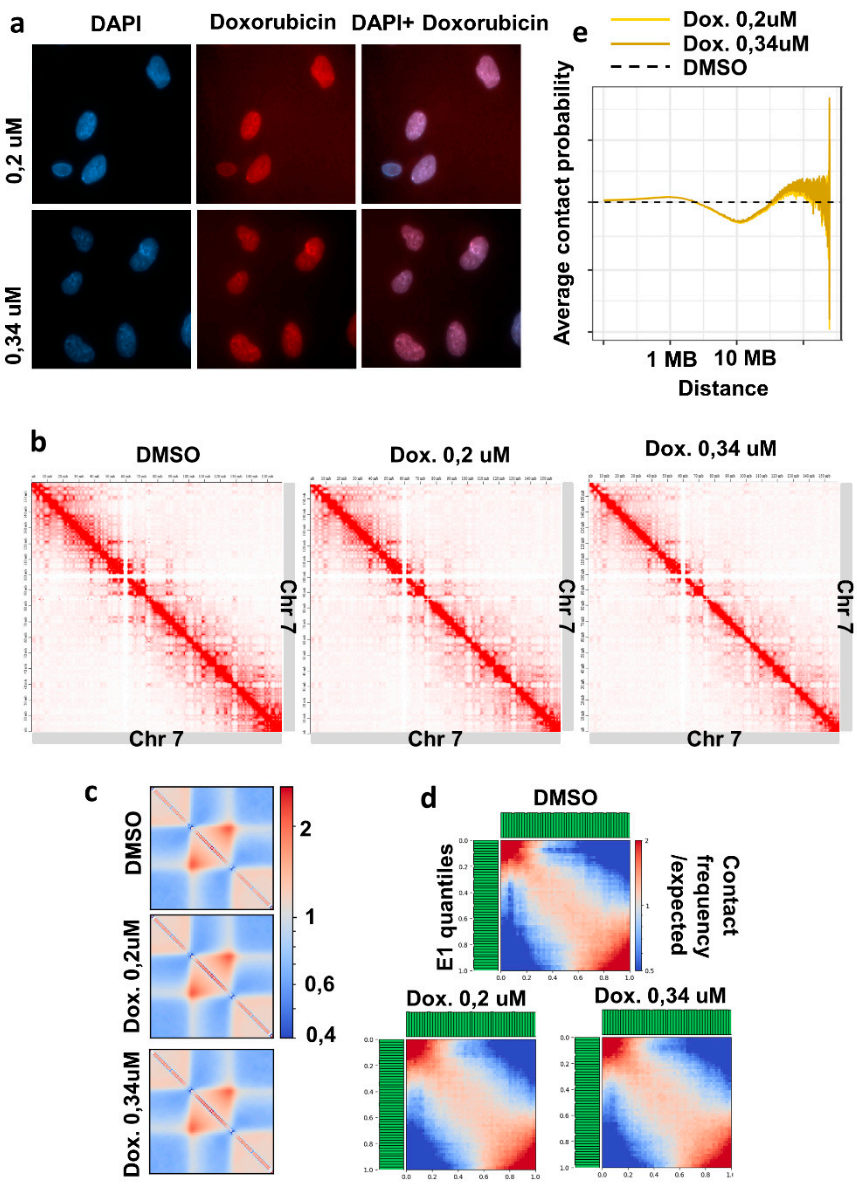

Figure S1. Whole-genome Hi-C analysis of the doxorubicin-treated cells.

a. Control of the nucleus penetration by doxorubicin. Doxorubicin autofluorescence (red) colocalizes with the DNA staining by DAPI (blue).

b. Examples of the Hi-C maps of the control cells (DMSO) and doxorubicin-treated cells. Chromosome 7 is shown.

c. Piled-up analysis of the TADs. Average TAD-structure is intact in doxorubicin-treated samples.

d. Aggregated analysis of compartments. Saddle plots for doxorubicin-treated cells and for the control are shown.

e. Distance decay plot of the Hi-C contacts of the doxorubicin-treated cells (yellow) contrasted to the control. Average contact probability by distance was calculated at 100kb resolution using FAN-C [1] and smoothed by fitting a cubic smoothing spline using the smooth.spline function in R.

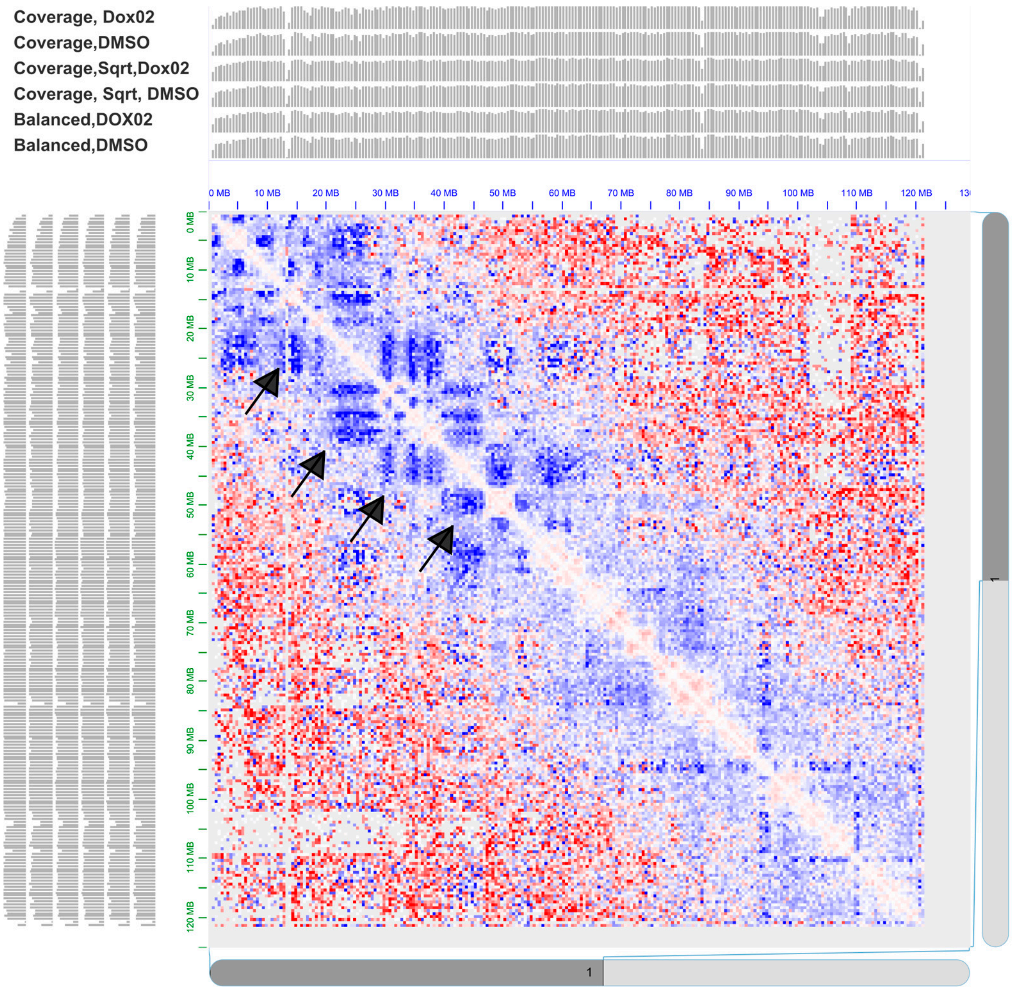

Figure S2. Hi-Coverage of the regions with decreased interactions is unchanged in doxorubicin-treated sample. Coverage of the map with or without normalization are shown. Regions of decreased interactions are visible in blue and are pointed at on the differential Hi-C map (observed/control).

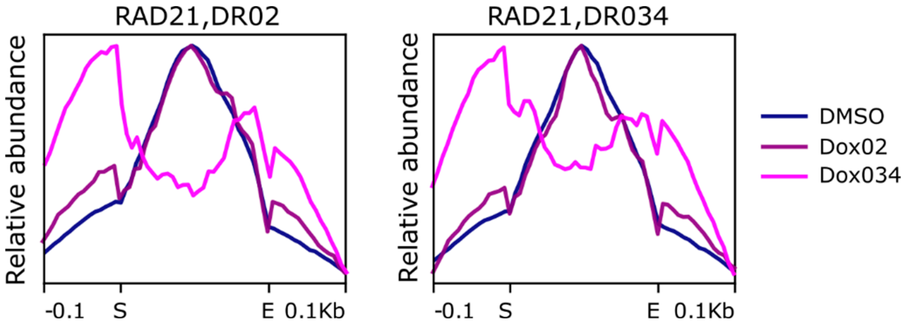

Figure S3. Relative RAD21 abundance around H3K27ac peaks. RAD21 distribution around H3K27ac peaks is shown. The distributions are calculated from the heat maps for the RAD21 signal around H3K27ac peaks. Results for one replicate of the control sample (DMSO) and two concentrations of doxorubicin are shown (DOX02 and DOX034). DR02 are the differential regions called from the Hi-C between control and cells treated with 200 nM of doxorubicin. DR034 are the differential regions between control and cells treated with 340 nM of doxorubicin. On the X axes “S” is a start coordinate of H3K27ac peak and “E” is an end coordinate of H3K27ac peak. Other replicates are shown on Fig. 5a.

To investigate changes in RAD21 binding following doxorubicin treatment, we conducted multiple replicates of ChIP-seq in two rounds of experiments. In the main figure (Figure 5), we presented heatmaps obtained from two biological replicates of DMSO treatment and two biological replicates of doxorubicin treatment. In this supplementary information section, we present results from an additional third replicate of DMSO treatment and two more replicates of doxorubicin treatment. Although the ChIP-seq results obtained for these additional replicates were noisier and of lower quality to construct heatmaps, we were able to generate meta-plots depicting the relative abundance of RAD21 around H3K27ac peaks (Fig. S3). Since our ChIP-seq was not calibrated, the meta-plots should not be compared quantitatively, but rather the distribution of the signal can be assessed. These meta-plots provide further support for our findings. Our results consistently demonstrate that doxorubicin treatment induces a redistribution of the cohesin complex around H3K27ac peaks within the differential Hi-C regions.

Top 10 biological processes for upregulated genes	% of all upregulated genes	FDR
response to stress	33	1.05E-07
apoptotic process	13	0.00000597
regulation of intracellular signal transduction	20	0.0000114
response to stimulus	54	0.0000165
cell death	14	0.0000183
regulation of cell death	18	0.0000187
programmed cell death	13	0.0000203
regulation of programmed cell death	17	0.0000218
regulation of apoptotic process	17	0.0000228
cellular response to stimulus	46	0.0000237

Top 10 biological processes for downregulated genes	% of all downregulated genes	FDR
cell cycle	50	2.94E-136
cell cycle process	43	8.98E-124
mitotic cell cycle	37	3.13E-116
mitotic cell cycle process	35	2.17E-112
cell division	23	7.99E-65
chromosome organization	30	1.73E-63
regulation of cell cycle	31	7.19E-59
regulation of cell cycle process	24	2.91E-51
cell cycle phase	16	6.24E-50
mitotic cell cycle phase	16	6.87E-50

Table S1.

The gene ontology shows that differential expression can be explained by the stress response and cell cycle inhibition.

Condition	ChIP-seq	Peaks underrepresented in treatment, FDR<0.05	Peaks overrepresented in treatment, FDR<0.05
Doxorubicin	CTCF	13	106
Doxorubicin	RAD21	0	0
ICRF193	CTCF	0	0
ICRF193	RAD21	0	0

Table S2.

Differential ChIP-seq peaks between doxorubicin-treated cells and control (DMSO) and ICRF193-treated cells and control.

1. Kruse, K.; Hug, C.B.; Vaquerizas, J.M. FAN-C: A Feature-Rich Framework for the Analysis and Visualisation of Chromosome Conformation Capture Data. *Genome Biol.* **2020**, *21*, 303, doi:10.1186/s13059-020-02215-9.
